# Effect of anti-inflammatory drugs on the storm of inflammatory factors in respiratory tract infection caused by SARS-CoV-2: an updated meta-analysis

**DOI:** 10.3389/fpubh.2023.1198987

**Published:** 2023-10-02

**Authors:** Zhiping Qin, Yongbiao Li, Wenjing Sun, Yangyang Lu, Nana Zhang, Rongfei Yang, Yiting Liu, Li Tang, Qingshan Liu

**Affiliations:** Key Laboratory of Ethnomedicine of Ministry of Education, School of Pharmacy, Minzu University of China, Beijing, China

**Keywords:** anti-inflammatory, SARS-CoV-2, COVID-19, efficacy, respiratory tract infection (RTI)

## Abstract

**Background:**

New reports suggest that anti-inflammatory drugs are widely used to treat respiratory tract infections caused by SARS-CoV-2. Anti-inflammatory drugs were the most frequently used treatment for the COVID-19-related cytokine storm in China. However, the efficacy of anti-inflammatory drugs has yet to be systematically analyzed, and clinicians are often uncertain which class of anti-inflammatory drug is the most effective in treating patients with respiratory tract infections caused by SARS-CoV-2, especially those with severe disease.

**Methods:**

From 1 October 2022, relevant studies were searched in the PubMed, Embase, Medline, Cochrane Library, and Web of Science databases. A total of 16,268 publications were retrieved and collated according to inclusion and exclusion criteria, and sensitivity analyses were performed using STATA 14 software. Publication bias was assessed using funnel plots and Egger’s test. Study quality was assessed using the PEDro scale, and the combined advantage ratio was expressed as a 95% confidence interval (CI). In total, 19 randomized controlled trials were included in the study. STATA 14 software was used for all random effects model analyses, and the results are expressed as relative risk ratios (RR) with 95% CI.

**Results:**

Quantitative analyses were performed on 14,514 patients from 19 relevant randomized controlled clinical trials. Pooled estimates (RR = 0.59, 95% CI 0.44–0.80) revealed that the use of anti-inflammatory drugs resulted in a significant reduction in mortality in patients with respiratory tract infection caused by SARS-CoV-2 compared with controls, and methylprednisolone (RR = 0.14, 95% CI 0.03–0.56) was more effective than other anti-inflammatory drugs. Anti-inflammatory drugs were effective in reducing mortality in critically ill patients (RR = 0.67, 95% CI 0.45–0.98) compared with non-critically ill patients (RR = 0.50, 95% CI 0.34–0.76); however, more clinical evidence is needed to confirm these findings.

**Conclusion:**

The use of anti-inflammatory drugs in patients with respiratory infections caused by SARS-CoV-2 reduces patient mortality, especially in severe cases. In individual studies, methylprednisolone was more effective than other drugs.

## 1. Introduction

Since the outbreak of coronavirus disease 2019 (COVID-19), more than 480 million cases have been confirmed by the World Health Organization. The mortality rate of COVID-19 is estimated to be 1.28% (1). Currently, respiratory infections caused by coronaviruses constitute a significant cause of global disease and public health burden. This has increased significantly since 2019, mainly due to the high morbidity, impact, and mortality of these infections (2), and researchers around the world are actively searching for safe and effective therapeutic drugs.

COVID-19 is caused by severe acute respiratory syndrome coronavirus 2 (SARS-CoV-2) (3), an enveloped, single-stranded RNA virus belonging to the β-coronavirus genus of the Coronaviridae family. SARS-CoV-2 is the third β-coronavirus to cause an outbreak in humans in the 21^st^ century (4). The inflammation of the lungs caused by the β-coronavirus is central to determining the severity of SARS-CoV-2 infection (5) and is critical in treating patients.

Recent studies have reported that SARS-CoV-2-induced hyperinflammatory syndrome exacerbates SARS-CoV-2 infection severity and increases mortality (6). The treatment of SARS-CoV-2 infection with anti-inflammatory drugs may alleviate the hyperinflammatory syndrome and reduce mortality and disease severity in the affected population. Anti-inflammatory drugs are commonly used to treat various chronic and infectious diseases, with varying mechanisms of anti-inflammatory action and clinical outcomes. The main drug classes used to manage SARS-CoV-2 infection are macrolide antibiotics, interleukin inhibitors, non-steroidal anti-inflammatory drugs, and biologics. Many studies have identified the cytokine storm as a significant cause of COVID-19 (2). The use of anti-inflammatory drugs to suppress the cytokine storm is crucial to reducing the spread of COVID-19.

## 2. Methodology

This study was conducted following the Preferred Reporting Items for Systematic Reviews and Meta-Analyses (PRISMA) guidelines (7), and the meta-analysis was registered with the International Prospective Register of Systematic Reviews (PROSPERO) on Oct 31, 2022 (registration number CRD42022371716). The subsequent retrieval of data was performed to minimize publication bias and increase the accuracy of the results.

### 2.1. Search strategy

Relevant searches were conducted on PubMed, Embase, Medline, Cochrane Library, and Web of Science databases using the following keywords: “anti-inflammatory,” “anti-inflammatory therapy,” “anti-inflammatory treatment,” “anti-inflammatory drugs,” “coronavirus disease,” “sars-cov-2 infection,” and “SARS-CoV-2 infection.” The search formula included the following terms: (“anti-inflammatory” OR “anti-inflammatory therapy” OR “anti-inflammatory treatment” OR “anti-inflammatory drugs”) AND (“coronavirus disease” OR “sars-cov-2 infection” OR “covid-19”). Randomized controlled studies were selected to filter the references and narrow the search.

### 2.2. Ethical approval

The respective ethics review boards approved all the included studies. Participant consent was obtained for each trial, and the local institutional review board requirements were met. The secondary data analyzed here are review studies that did not require approval for registration by the relevant institutional review boards or ethics committees. We collated the study data for analysis after registration with PROSPERO.

### 2.3. Selection of research criteria

The inclusion and exclusion criteria for this study were developed by two authors (QZP and LYB). The inclusion criteria were (a) adult cases of SARS-CoV-2 infection, (b) participants treated with anti-inflammatory drugs, (c) placebo or standard care controls, (d) death as one of the study outcomes, and (e) randomized controlled studies. The exclusion criteria were (a) no control group, (b) insufficient sample size, (c) a disease other than not new coronary pneumonia, (d) a study design other than a randomized controlled trial, (e) missing necessary data (e.g., number of participants in the study, number of deaths), and (f) no anti-inflammatory drug treatment.

### 2.4. Quality assessment and risk of bias

The quality of the included articles was evaluated using the PEDro Scale for Rating Quality of Randomized Controlled Trials. Articles were scored for inclusion, and three authors (QZP and LYB) performed the article scoring separately. Articles were classified into three categories according to the score: high quality (8–10), acceptable quality (5–8, 11), or poor quality (0–5). A funnel plot was used for qualitative analysis of publication bias.

### 2.5. Data extraction

The data included in this study were extracted into an Excel spreadsheet by three authors (QZP and LYB). The data included the article title, country, author, journal name, year of publication, impact factor, DOI number, article abstract, study results, type of study design, leading outcome indicators, total number of participants, number of people in the intervention group, number of people in the control group, number of deaths in the intervention group, and number of deaths in the control group.

### 2.6. Statistical analysis

All data were analyzed using STATA 14 software package. A random-effects model was used. Overall estimates were calculated as risk ratios (RR) with 95% confidence intervals (CI) using the I^2^ statistic and L’Abbé plots to indicate heterogeneity in the included data.

### 2.7. Sensitivity analysis

The effect of each study analysis on the results was removed to produce a sensitivity analysis graph for the relevant sensitivity analysis.

### 2.8. Subgroup analysis

Subgroup analysis was performed according to disease severity (severe vs. non-severe) and anti-inflammatory drug classes (interleukin inhibitors, corticosteroids, natural drugs and their extracts, or combination drugs) grouped in the study population. The criteria for distinguishing severe versus non-severe patients included the type of patient (which was reported by some of the studies), while those not labeled were classified according to the following criteria: (a) respiratory distress (respiratory rate ≥ 30 breaths/min); (b) resting state finger oxygen saturation ≤ 93%, arterial partial pressure of oxygen (PaO_2_)/inhaled oxygen concentration (FiO_2_) ≤300 mmHg; (c) progressive worsening of clinical symptoms; (d) lung imaging showing the significant progression of >50% of the lesion within 24–48 h; and (e) respiratory failure and the need for mechanical ventilation. Subgroup analysis was performed after examination of the overall data, with or without hospitalization included in the meta-analysis, which was agreed upon before the start of the study.

## 3. Results

### 3.1. Database search

**Figure 1:**
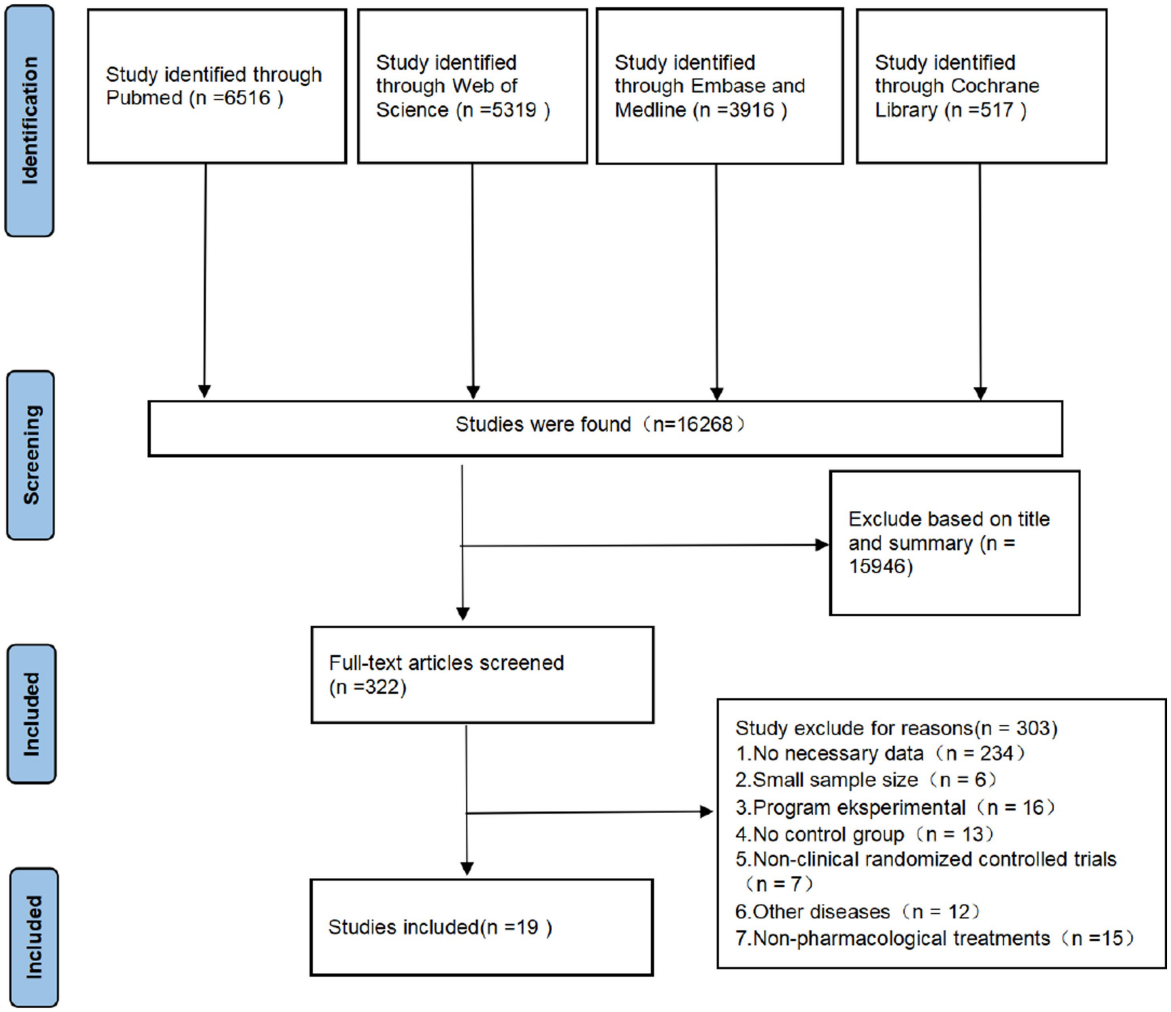
Research process according to the PRISMA flowchart.

The initial literature search was conducted on Nov 10, 2022, using the PubMed, Embase, Medline, Cochrane Library, and Web of Science databases. The initial search identified 16,268 studies, and after reviewing the titles and abstracts, 322 were downloaded for full-text information retrieval, and 19 were identified for inclusion in the analysis. All 16 studies were randomized controlled clinical studies (8–26). One study was from France, two were from the United States, four were from Iran, three were from the UK, one was from Italy, one was from China, one was from Indonesia, three were from Brazil, one was from Greece, one was from Argentina, and one was from Egypt. The flow chart of the search process is shown in Figure 1.

### 3.2. Basic characteristics of the included studies

The essential characteristics of the included studies are listed in Table 1. The 19 included studies included a total of 14,514 participants, and all were randomized controlled studies, including one triple-blind controlled trial, 10 double-blind controlled trials, one single-blind controlled trial, five open-label trials, and two randomized prospective clinical trials. All studies reported the expected outcomes according to pre-defined experimental protocols, where indicators related to mortality or the number of deaths were included in the primary or secondary outcomes. Adverse effects were reported in nine studies, five included patients with severe respiratory tract infection caused by SARS-CoV-2, three were conducted in a community setting, six used a placebo as a control group, four used corticosteroids, five used interleukins and angiotensin inhibitors, one used antibiotics, one used statins, and five used natural drugs and their extracts as anti-inflammatory drugs. Combinations of drugs appeared in three studies, with relevant subgroup analyses.

**Table 1:** Basic characteristics of the included studies.

Drugs/Year	IF	Country	Doi	Study design	Type of Disease disease	NO.	Start time	Age*	Evaluation interval(day)
Hydrocortisone. 2020	45.54	France	10.1001/jama.2020.16761	Randomized, double-blind controlled trials	COVID-19	149	2020.3.7	62.2	28
Azithromycin+Hydroxychloroquine. 2022	33.795	USA	10.1183/13993003.00752-2021	Placebo-controlled, double-blind, randomized multicenter trial	COVID-19	117	2020.4.6	65	15
Lenzilumab. 2021	30.7	USA	10.1016/s2213-2600(21)00494-x	Phase 3 randomized, double-blind, placebo-controlled trial	COVID-19	520	2020.5.5	61	28
Anakinra. 2021	53.44	Greece	10.1038/s41591-021-01499-z	A double-blind, randomized controlled trial	COVID-19	594	2020.12.23	61.9	28
Colchicine. 2021	30.7	Canada	10.1016/s2213-2600(21)00222-8	Phase 3, randomized, double-blind, adaptive, placebo-controlled, multicenter trial	COVID-19	4,488	2020.3.23	54	30
Nano-curcumin. 2021	4.932	Iran	10.1016/j.intimp.2020.107088	Randomized, double-blind, placebo-controlled	COVID-19	40	NA	NA	14
Tocilizumab. 2021	93.333	Brazil	10.1136/BMJ.n84	Randomized, open-label trials	COVID-19	129	2020.5.8	57	15
Dexamethasone. 2020	70.67	UK	10.1056/NEJMoa2021436	Randomized controlled open-label trials	COVID-19	6,425	2020.3.19	66.1	28
Methylprednisolone. 2020	12.339	Iran	10.1183/13993003.02808-2020	Single-blind, two-arm parallel, randomized controlled trial	COVID-19	68	2020.4.20	58.5	10
Umbilical cord mesenchymal stromal cells. 2021	6.94	Indonesia	10.1002/sctm.21-0046	Multicenter, double-blind, randomized clinical trial	COVID-19	40	2020.5.1	NA	30
EPP-AF®. 2021	6.529	Brazil	10.1016/j.biopha.2021.111526	Single-center, open-label, randomized, controlled trial	COVID-19	124	2020.6.3	50	28
Xuebijing. 2021	1.314	China	10.1016/j.eujim.2021.101305	Single-center, prospective, randomized, double-blind trial	COVID-19	57	2020.2.16	NA	28
Adalimumab. 2021	4.932	Iran	10.1016/j.intimp.2021.107961	Randomized prospective controlled trials	COVID-19	68	NA	NA	11
Budesonide. 2021	79.321	UK	10.1016/s0140-6736(21)01744-x	Randomized, controlled, open-label, adaptive platform trials	COVID-19	1856	2020.4.2	64.2	28
Azithromycin. 2021	60.392	UK	10.1016/s0140-6736(21)00461-x	Randomized, controlled, open-label, adaptive platform trials	COVID-19	1,323	2020.4.2	60.7	28
Quercetin. 2021	2.466	Italy	10.2147/IJGM.S318949	Prospective, randomized, controlled, and open-label pilot studies	COVID-19	42	2020.12	49.3	14
Glycyrrhizin+Boswellic acids. 2022	3.238	Egypt	10.1002/sctm.21-0046	randomized, double-blind, placebo-controlled, single-center trial	COVID-19	50	202.03.01	61.3	14
Telmisartan. 2021	17.033	Argentina	10.1016/j.eclinm.2021.100962	parallel-group, randomized, two-arm, open-label, adaptive, multicenter superiority trial	COVID-19	158	2021.06.18	65.3	30
Atorvastatin. 2022	NA	Iran	10.2174/2772434417666220902153823	randomized, parallel-design, triple-blind, and placebocontrolled clinical trial	COVID-19	40	2022.01.01	54.6	14

### 3.3. Inclusion of study outcomes and article quality

The primary outcomes and outcome indicators of the included studies are listed in Table 2. The randomized controlled studies were evaluated using the PEDro scale, and the results of the article quality assessment are presented in Table 2.

**Table 2:** Characteristics of the included study outcomes and quality of articles.

Drugs/Year	Study design	Primary outcome measures	Samples (DS/Control)	Gender (% Male) (DS/Control)	Mean Age (DS/Control)	Route of administration	Treatment information collection	OR/RR/HR	95%CI	Outcomes	PEDro score
Hydrocortisone. 2020	Randomized, double-blind controlled trials	Death at 21 days or continued dependence on mechanical ventilation or high-flow oxygen therapy.	76/73	54/50	63.1/66.3	Intravenous injection	Case records	NA	−24.9 ~ 7.7	Low-dose hydrocortisone did not significantly reduce treatment failure (defined as death or sustained respiratory support) at day 21.	8
Azithromycin+Hydroxychloroquine. 2022	Placebo-controlled, double-blind, randomized multicenter trial	Days to survive and discharge within 14 days of randomization (DAOH).	61/56	59/52	68/63	NA	NA	1	NA	The combination of azithromycin and hydroxychloroquine did not improve survival or length of hospital stay in patients with COVID-19.	10
Lenzilumab. 2021	Phase 3 randomized, double-blind, placebo-controlled trial	IMV, death rate, or recovery on day 28。	236/243	65/65	61/61	Intravenous drip	NA	1.54	1.02 ~ 2.32	Renzilumab improves survival in hospitalized adults with COVID-19 pneumonia and reduces the risk of IMV.	10
Anakinra. 2021	Double-blind randomized controlled trial	RT-PCR negative rate.	189/405	58.3/57.1	62/61.5	Take orally	Case records	0	0.26 ~ 0.49	Guided by suPAR levels, early initiation of Anakinra for hospitalized patients with moderate and severe COVID-19 significantly reduced the risk of worsening clinical outcomes on day 28.	8
Colchicine. 2021	Phase 3, randomized, double-blind, adaptive, placebo-controlled, multicenter trial	Death or hospitalization due to COVID-19 infection within 30 days of randomization.	2235/2253	44.6/47.5	53/54	Take orally	Follow-up for 30 days	0.79	0.59 ~ 0.94	Mid-colchicine reduces compounded mortality or hospitalization in patients with PCR-confirmed COVID-19.	10
Nano-curcumin. 2021	Randomized, double-blind, placebo-controlled	The amount of inflammatory factor expression	20/20	77/80	53.3/51.4	Injection	Blood sampling	NA	NA	Nano curcumin can improve clinical presentation and overall recovery.	7
Tocilizumab. 2021	Randomized, open-label trials	15 days of mechanical ventilation or death.	65/64	68/69	57.4/57.5	Intravenous injection	Electronic medical case report form	1.54	0.66 ~ 3.66	Tocilizumab plus standard therapy is ineffective in patients with severe or critical COVID and may increase mortality.	7
Dexamethasone. 2020	Randomized controlled open-label trials	28-day mortality.	2104/4321	64/64	66.9/65.8	Intravenous injection/Take orally	Web-based case report form collects baseline data/online follow-up form	0.83	0.75 ~ 0.93	Treatment with dexamethasone at a dose of 6 mg reduces 28-day mortality in Covid-19 patients who are receiving respiratory support.	7
Methylprednisolone. 2020	Single-blind, two-arm parallel, randomized controlled trial	Time to clinical improvement or death.	34/28	62.9/70.6	55.8/61.7	Intravenous injection	Case Report Form/Excel Database	0.293	0.154 ~ 0.555	Methylprednisolone pulse may be used as an effective treatment for hospitalized patients with severe COVID-19 lung stage.	8
Umbilical cord mesenchymal stromal cells. 2021	Multicenter, double-blind, randomized clinical trial	Mortality and length of ventilator use.	20/20	75/75	18–95	Intravenous drip	Case report form	NA	NA	Intravenous UC-MSCs as adjunctive therapy for critically ill patients with COVID-19 have improved survival.	9
EPP-AF®. 2021	Single-centre, open-label, randomized, controlled trial	Length of hospital stay or oxygen therapy dependence.	82/42	70.7/66.7	49.2/51.6	Take orally	Follow	NA	−6.23 ~ −0.07/−7 ~ −0.09	Propolis has had clinical benefits for hospitalized COVID-19 patients, reducing hospital stays.	10
Xuebijing. 2021	Single-center, prospective, randomized, double-blind trial	Peripheral blood lymphocyte and IL-6 levels on days 1, 7, and 14 of enrollment.	29/28	NA	60.26/50.35	Intravenous injection	Sample collection	NA	NA	Xuebijing injection may suppress the cytokine storm in severe COVID-19 patients by regulating the secretion of pro- inflammatory cytokine IL-6, IL-8 and TNF -α. However, Xuebijing did not significantly reduce the 28-day mortality.	7
Adalimumab. 2021	Randomized prospective controlled trials	Invasive mechanical ventilation, the need for intensive care unit (ICU) admission, and mortality are required.	34/34	64.7/52.94	53.15/56.12	Subcutaneous injection	Medical records	NA	NA	Adalima combined with redivir and dexamethasone is ineffective in the treatment of severe new coronary pneumonia.	5
Budesonide. 2021	Randomized, controlled, open-label, adaptive platform trials	Hospitalization or death or duration of illness within 28 days.	787/1069	48/48	64.7/63.8	Inhale	Online daily symptom diary/phone callback	0.75	1.19 ~ 5.12	Budesonide is safe and effective for people with COVID-19 who are at increased risk of adverse outcomes in the community.	7
Azithromycin. 2021	Randomized, controlled, open-label, adaptive platform trials	First recovery, hospitalization, or death within 28 days of randomization	540/875/850	43/44	60.9/60.5	Take orally	Daily online diary	1.12	0.91 ~ 1.38	Routine use of azithromycin does not reduce recovery time or hospitalization risk in patients with suspected COVID-19 in the community.	7
Quercetin. 2021	Prospective, randomized, controlled, and open-label pilot studies	RT-PCR negative time, inflammatory factor indicators, need for hospitalization.	21/21	47.62/47.62	42.5/56.2	Take orally	Medical records	NA	NA	QP shortens the time for molecular tests to change from positive to negative, reducing the severity of symptoms and negative predictors of new coronary pneumonia.	5
Glycyrrhizin+Boswellic acids. 2022	randomized, double-blind, placebo-controlled, single-center trial	RT-PCR	25/25	11/12	60/62.5	Take orally	Standardized questionnaires	NA	NA	In conclusion, this safe, inexpensive, antiviral, immunomodulating and anti-inflammatory combination may be considered for use in mild to moderate infections of SARS-CoV-2 or COVID-19 variants.	7
Telmisartan. 2021	parallel-group, randomized, two-arm, open-label, adaptive, multicenter superiority trial	RT-PCR	78/80	49/35	63.7/66.9	NA	Build the database	NA	3.44–1.30	Our study suggests that the ARB telmisartan, a widely used antihypertensive drug, is safe and could reduce morbidity and mortality in hospitalized patients infected with SARS -CoV-2 by anti-inflammatory effects.	8
Atorvastatin. 2022	randomized, parallel-design, triple-blind, and placebocontrolled clinical trial	RT-PCR/CT abnormalities	20/20	6/8	51.5/57.7	Take orally	Case records	NA	NA	Atorvastatin significantly reduces supplemental oxygen need, hospitalization duration, and serum hs-CRP level in mild to moderate hospitalized COVID-19 patients.	9

### 3.4. Use of anti-inflammatory drugs in patients with respiratory infections caused by SARS-CoV-2

**Figure 2:**
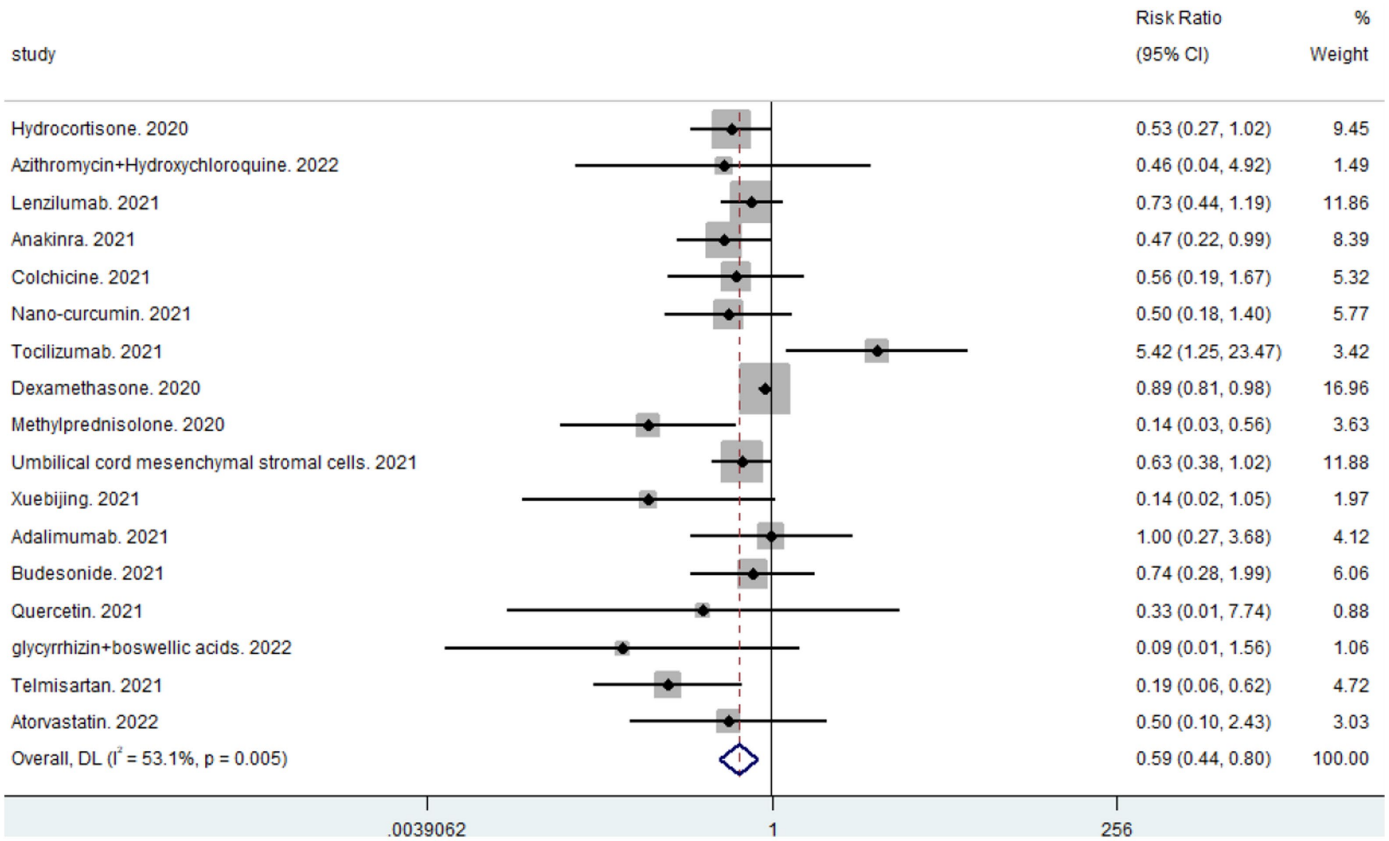
Forest plots for integrated analysis using random effects models.

**Figure 3:**
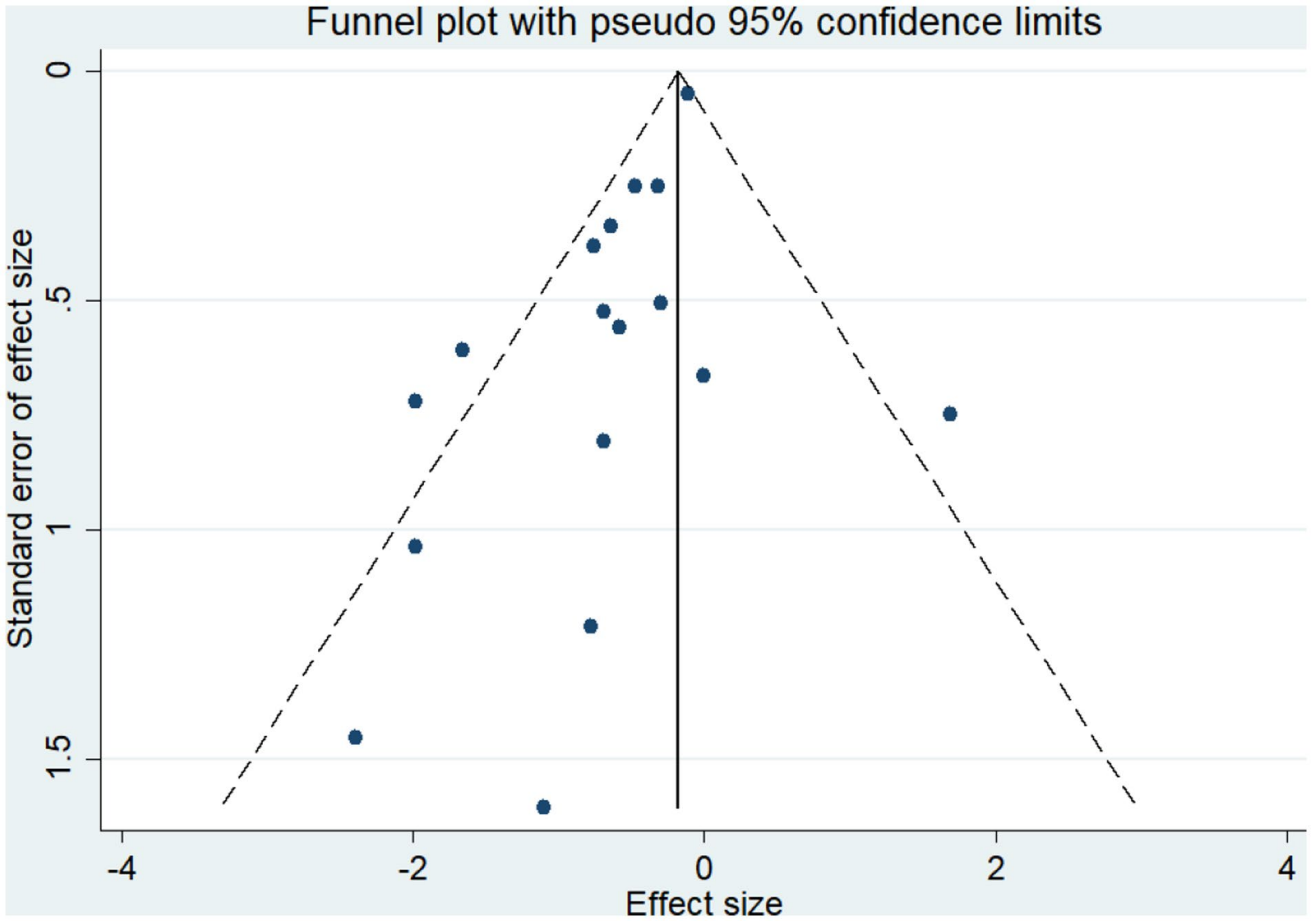
Funnel plot for qualitative publication bias.

A total of 19 studies, including 14,514 participants, were included. Of the 14,514 patients, 5,995 received anti-inflammatory drugs and 8,288 received standard care, usual care, or placebo, with a post-combination RR of 0.59 (95% CI 0.44–0.80). From the analysis (Figure 2), it can be concluded that the use of anti-inflammatory drugs significantly reduced mortality in patients with respiratory infections caused by SARS-CoV-2 compared with the control groups. The funnel plot (Figure 3) did not indicate significant symmetry and was derived by Egger’s test (Table 3), with a value of *p* = 0.008 (<0.05), indicating publication bias.

**Table 3:** Egger’s test results.

Std_Eff	Coeff.	Standard error.	*t*	P > |t|	[95% Conf. Interval]	
slope	−0.0726519	0.0622786	−1.17	0.262	−0.2053955	0.0600917
bias	−1.042555	0.3417146	−3.05	0.008	−1.770902	−0.3142076

### 3.5. Sensitivity analysis

**Figure 4:**
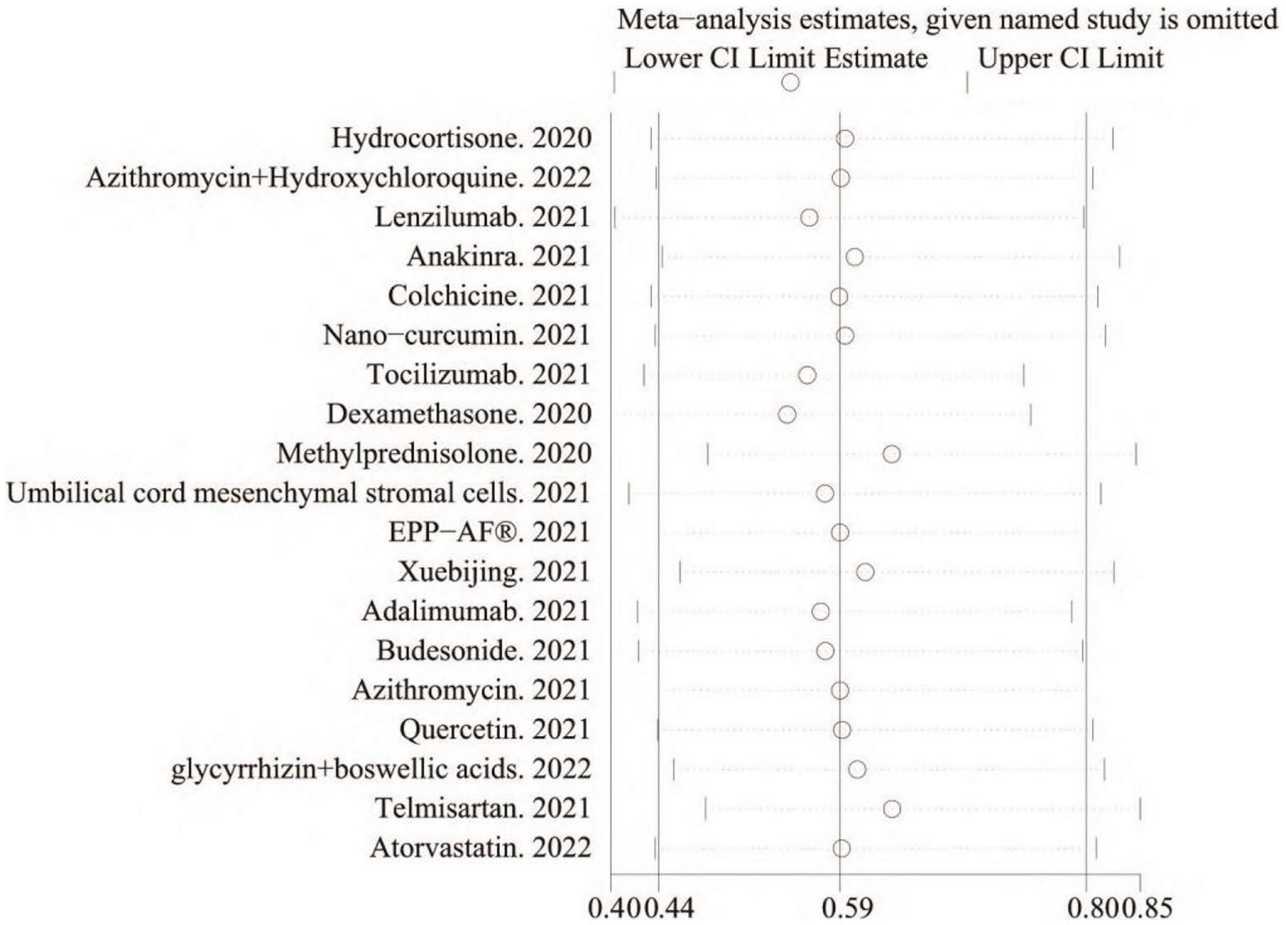
Incorporation of data sensitivity analysis chart.

Correlation sensitivity analysis was performed to evaluate the impact of the included studies on the analysis of the outcomes. Of the 19 included studies, the effect on the combined RR was greater after removing the studies that used dexamethasone, methylprednisolone, and temafloxacin, all of which had either too large or too small sample sizes, indicating that the sample size had a more significant effect on the experimental results (Figure 4).

### 3.6. Heterogeneity analysis

The heterogeneity of the results was analyzed using the I^2^ statistic (Figure 2) and L’Abbé plots (Figure 4). An I^2^ value of 53.1% and a concentrated L’Abbé plot graph indicated moderate heterogeneity of the data from the included studies, which could be combined for analysis.

### 3.7. Subgroup analysis

Subgroup analyses were conducted to examine the impact of anti-inflammatory drugs on the number of deaths in critically ill versus non-critically ill patients. Subgroup analyses of different classes of anti-inflammatory drugs were conducted to determine which class of anti-inflammatory drugs had the most significant impact on mortality in patients with respiratory tract infections caused by SARS-CoV-2.

### 3.8. Subgroup analysis of critically Ill and non-critically Ill patients

**Figure 5:**
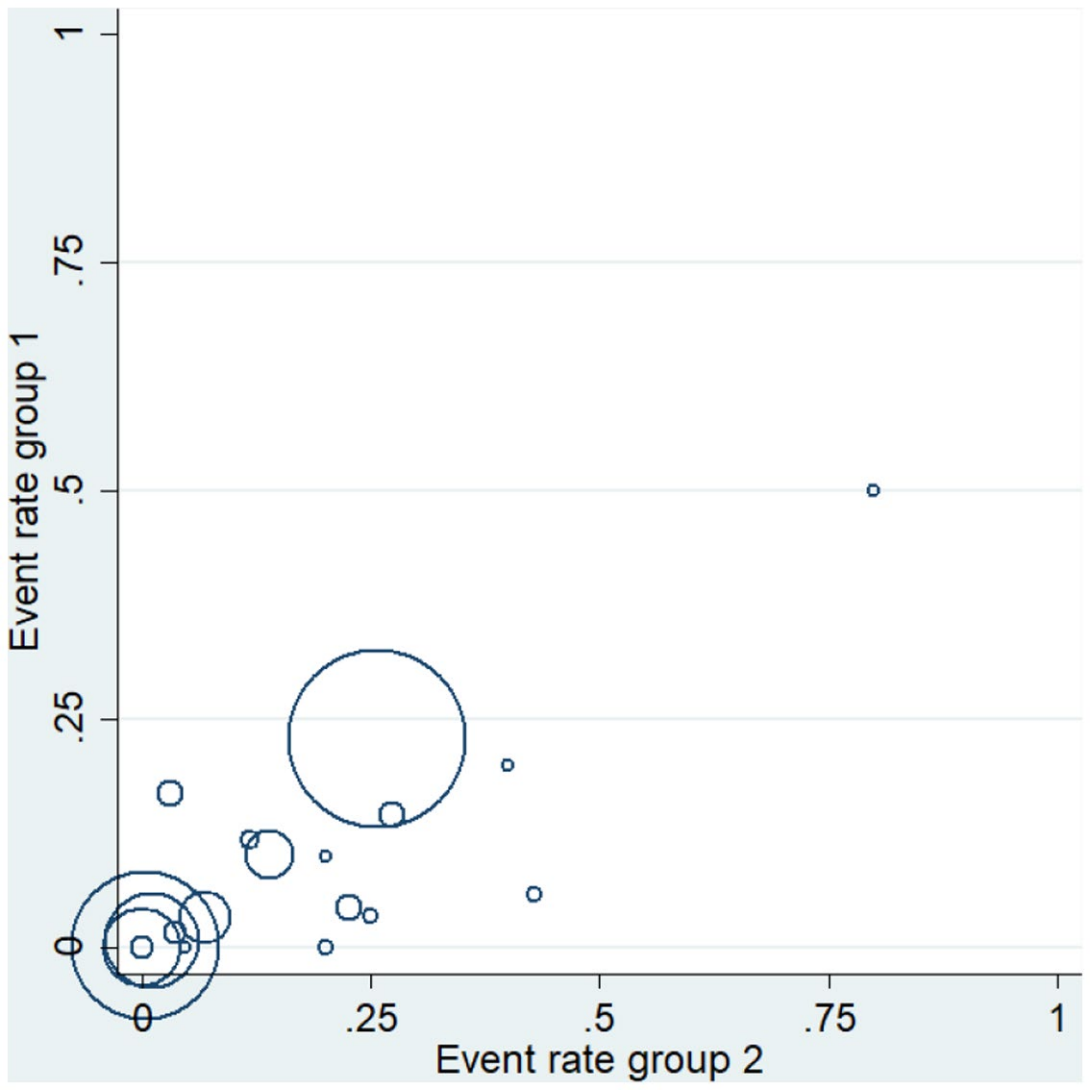
Heterogeneity analysis: L’Abbé diagram.

Subgroup analyses were performed to evaluate the effects of the anti-inflammatory drugs included in the studies on the mortality of patients with severe versus non-severe COVID-19 (combined critically ill: RR 0.50, 95% CI 0.34–0.76; combined non-critically ill: RR = 0.67, 95% CI 0.45–0.98). From a comparison of the combined results (Figure 5), it can be concluded that anti-inflammatory drugs had a more significant impact on mortality in critically ill than in non-critically ill patients. Anti-inflammatory drugs significantly reduced mortality in patients with severe respiratory tract infections caused by SARS-CoV-2.

### 3.9. Analysis of anti-inflammatory drug subgroups

**Figure 6:**
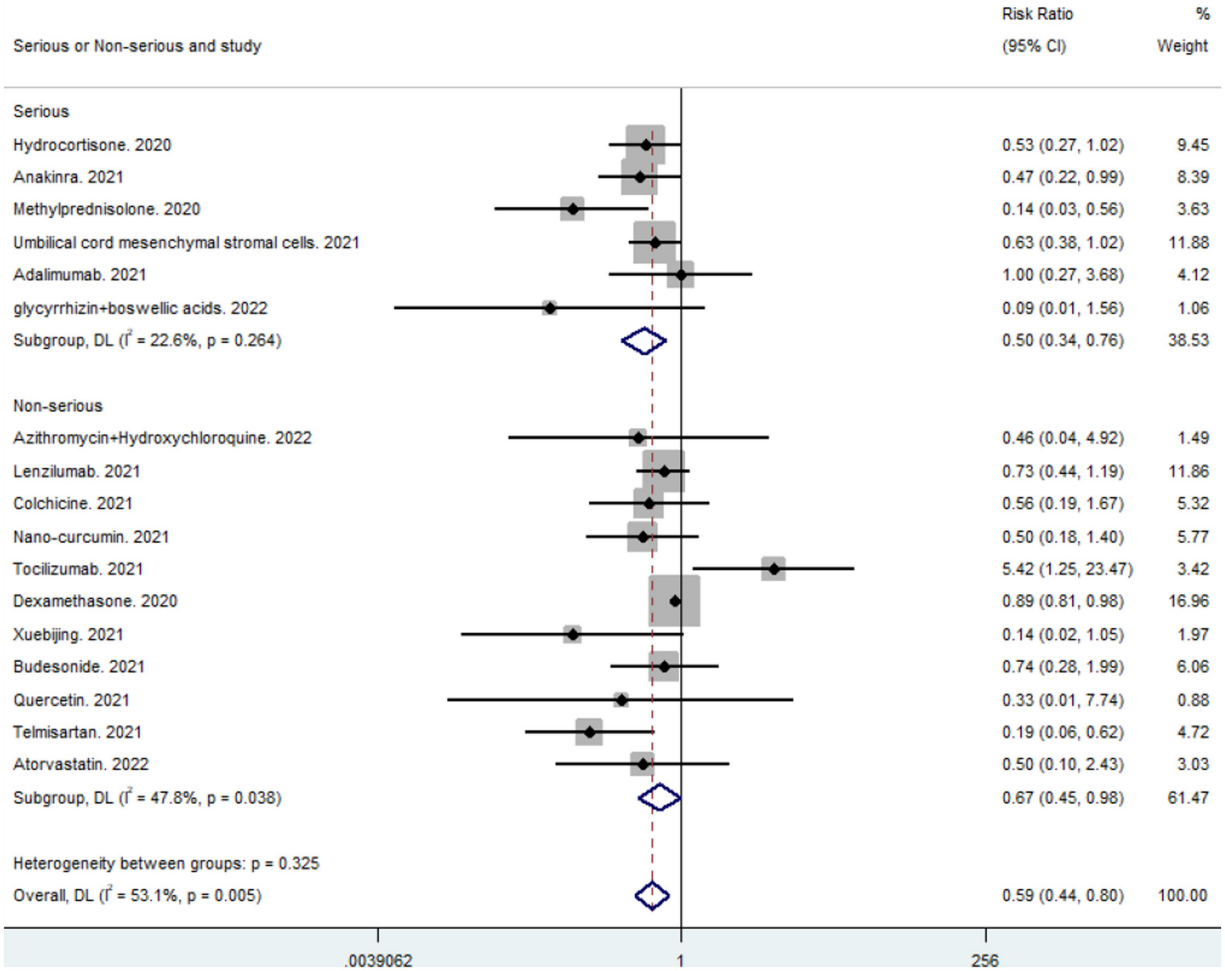
Results of subgroup analysis of critically ill and non-critically ill patients.

**Figure 7:**
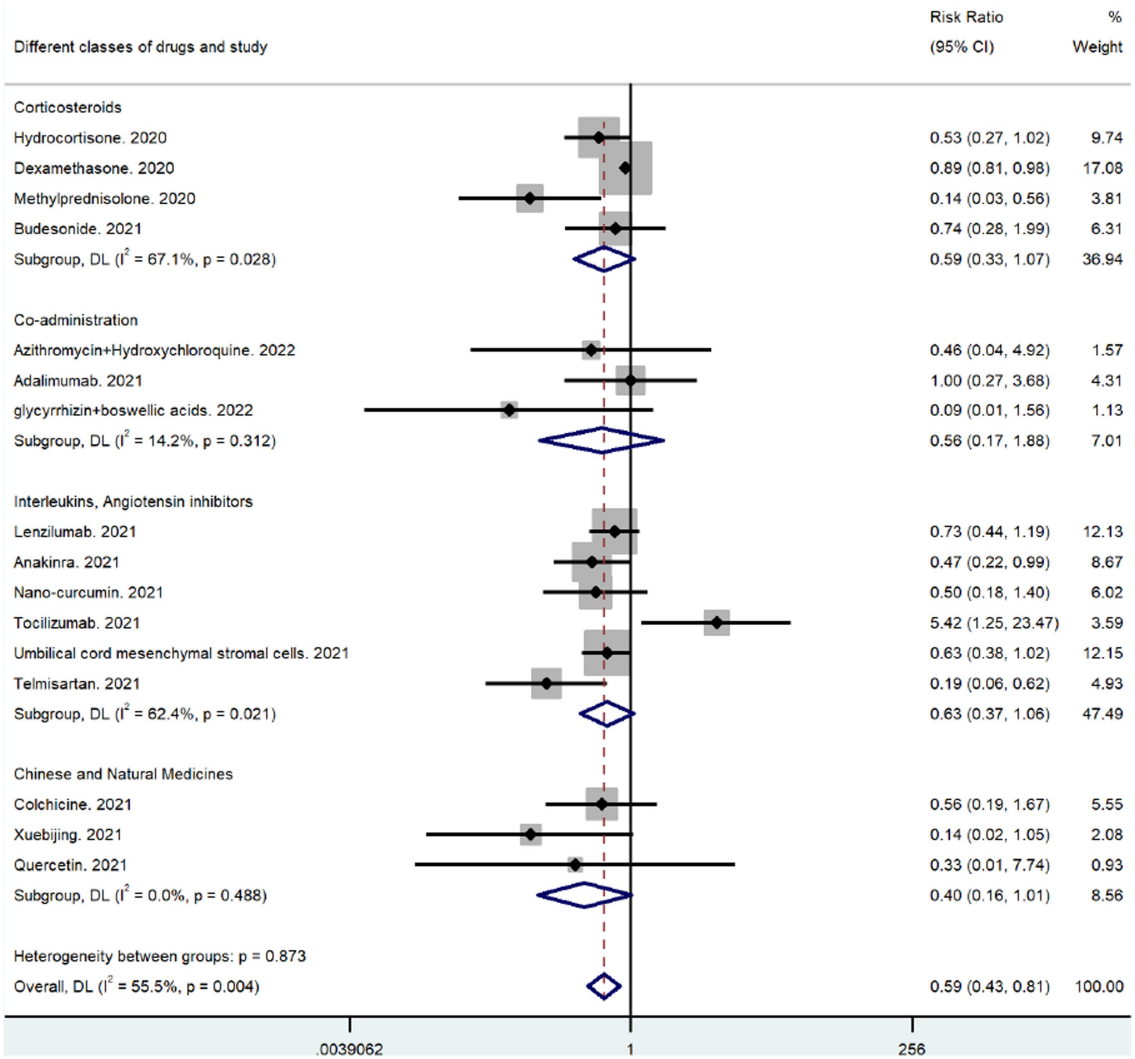
Results of subgroup analysis for different types of drugs.

The only antibiotic anti-inflammatory drug used was the macrolide azithromycin. The relevant studies reported no subject deaths during their study periods, and the other medicines were categorized for subgroup analysis. After combining the corticosteroid results, the RR was 0.59 (95% CI 0.33–1.07), indicating that corticosteroids significantly reduced mortality in patients with respiratory infections caused by SARS-CoV-2 (Figure 6). As shown in Figure 6, the combination of drugs greatly affected coronavirus-induced respiratory infections; however, the effect was not statistically significant (*p* = 0.312). Because the mechanism of anti-inflammatory action of umbilical cord MSCs is like that of interleukin inhibitors, they were classified in the broad category of interleukin inhibitors. The RR for the combined outcome of the interleukin inhibitor class was 0.63 (95% CI 0.37–1.06), which indicated reduced mortality in patients with coronavirus-induced respiratory infection following treatment (Figure 6). The overall effect was greater than that of corticosteroids and interleukin inhibitors (Figure 6) (RR = 0.40, 95% CI 0.16–1.01) for the combined post-outcome RR of natural drugs and their extracts. However, the results must be further evaluated as they were not statistically significant (*p* = 0.488). The comparative results suggest that corticosteroids significantly reduce mortality in patients with a coronavirus-induced respiratory infection, with the corticosteroid methylprednisolone (RR = 0.14, 95% CI 0.03–0.56) being the most effective and a potential focus of future clinical studies (see Figure 7).

## 4. Discussion

Anti-inflammatory drugs are crucial in reducing mortality in patients with SARS-CoV-2 infection, especially in those with severe disease. In this meta-analysis, the overall effectiveness of several drugs was evaluated according to patient mortality. This meta-analysis aimed to examine the association between the use of anti-inflammatory drugs and mortality and to identify the anti-inflammatory drugs that are the most effective in reducing mortality in patients with SARS-CoV-2 infection, especially in severely ill patients. Our results suggest that several anti-inflammatory drugs significantly reduced mortality in patients with severe or non-severe SARS-CoV-2 infections, which is inconsistent with previous meta-analyses. The results of the subgroup analysis revealed that not all anti-inflammatory drugs were equally effective. Corticosteroids and natural anti-inflammatory drugs were more effective than other anti-inflammatory drugs in treating patients with severe respiratory tract infection caused by SARS-CoV-2, and methylprednisolone and a combination treatment comprising glycyrrhizin, boswellic acids, and Xuebijing was more effective in individual studies; however, the effect of natural anti-inflammatory drugs and their combination was not statistically significant and should be further analyzed in future studies.

A meta-analysis by Thakur et al. (27) on corticosteroids revealed that their use in patients hospitalized with SARS-CoV-2 infection reduced mortality. A meta-analysis by Li et al. (28) suggested that corticosteroids significantly reduced mortality in severe SARS-CoV-2 infection, especially when administered early. A recent meta-analysis by Liu et al. (29) reported that corticosteroids were more effective and safer than standard care for treating SARS-CoV-2 infection. The results of these meta-analyses are consistent with our results and strongly suggest that corticosteroids play a crucial role in treating patients with SARS-CoV-2 infection, especially those who are severely ill.

In the meta-analysis of interleukin inhibitors, studies on tocilizumab predominated. A systematic review and meta-analysis by Muhammad et al. (30) revealed that the addition of tocilizumab to the standard care group reduced mortality and the need for mechanical ventilation in patients with severe SARS-CoV-2 infection; however, this result was not confirmed in clinical trials. Our meta-analysis revealed that tocilizumab did not reduce mortality in patients with SARS-CoV-2 infection and may have instead increased mortality. In a meta-analysis, Yu et al. (31) reported that tocilizumab treatment produced promising results in reducing 28-day mortality in patients with moderate-to-severe SARS-CoV-2 infection, without serious adverse events.

In a meta-analysis related to the macrolide antibiotic azithromycin, Ayerbe et al. (32) reported that it was not effective in reducing the mortality of patients with respiratory tract infection caused by SARS-CoV-2. A meta-analysis by Kamel et al. (33) concluded that due to the lack of efficacy and the potential risk of bacterial resistance, the routine use of azithromycin in patients with respiratory tract infections caused by SARS-CoV-2 was not justified and did not meet the current required clinical benefit. Furthermore, the results of the randomized controlled trials included in this meta-analysis did not indicate that azithromycin produced any benefit in mortality reduction in patients with respiratory tract infection caused by SARS-CoV-2.

Analysis of the combination of several classes of anti-inflammatory drugs revealed that polytherapy was more beneficial than monotherapy in reducing mortality in patients with respiratory tract infections caused by SARS-CoV-2. A meta-analysis by Lim et al. (34) concluded that the combination of tocilizumab and corticosteroids was more effective than either of these drugs alone. This result was confirmed by a meta-analysis by Moosazadeh et al. (35). Moreover, Chinese policies emphasize that most critically ill patients with novel coronavirus pneumonia will develop respiratory failure and that the combination of appropriate respiratory support (including invasive and non-invasive mechanical ventilation) and clinical extracorporeal membrane lung oxygenation with pharmacological therapy is the clinical guideline for the treatment of critically ill patients. In addition, the results of the meta-analysis suggest that the natural drugs available for the treatment of SARS-CoV-2 are less relevant for the treatment of SARS-CoV-2 with natural drugs and their extracts. However, our meta-analysis revealed that the natural drug extract Xuebijing significantly reduced mortality in patients with respiratory tract infection caused by SARS-CoV-2, which should be analyzed in depth in future clinical trials.

In conclusion, anti-inflammatory drugs can significantly reduce mortality in patients with respiratory infections caused by SARS-CoV-2, especially in critically ill patients. Furthermore, the use of anti-inflammatory drugs in combination with corticosteroids or respiratory support at the time of treatment can dramatically reduce mortality in critically ill patients. In individual studies, methylprednisolone was significantly more effective than other anti-inflammatory drugs. However, this meta-analysis only included clinical randomized controlled trials related to respiratory infections caused by SARS-CoV-2 in the last 2 years. More relevant clinical data are needed from future experimental and clinical studies.

## 5. Limitations

This study has several limitations. Only randomized controlled trials with control groups were considered, limited databases were used, and only articles published in English were considered.

## Author contributions

QL and LT conceived the article, while ZQ designed and contributed equally to the article. ZQ, YoL, and WS performed the preliminary literature screening. ZQ and YoL received all the data used in the article. ZQ, YaL, NZ, RY, and YiL were involved in the analysis and interpretation of the data. ZQ drafted and revised all the relevant manuscripts. All authors contributed to the article and approved the submitted version.

## Funding

This study was supported by the National Natural Science Foundation of China (82174085).

## Conflict of interest

The authors declare that the research was conducted in the absence of any commercial or financial relationships that could be construed as a potential conflict of interest.

## Publisher’s note

All claims expressed in this article are solely those of the authors and do not necessarily represent those of their affiliated organizations, or those of the publisher, the editors and the reviewers. Any product that may be evaluated in this article, or claim that may be made by its manufacturer, is not guaranteed or endorsed by the publisher.
