# Supplementary material for: Effect of anti-inflammatory drugs on the storm of inflammatory factors in respiratory tract infection caused by SARS-CoV-2: an updated meta-analysis
Source: Front Public Health. 2023 Oct 2;11:1198987. doi: 10.3389/fpubh.2023.1198987 (PMC10619852; doi:10.3389/fpubh.2023.1198987)

Specific search form and specific search results:

1. Pubmed：
2. Search formula：('anti-inflammatory' OR 'anti-inflammatory therapy' OR 'anti-inflammatory treatment' OR 'anti-inflammatory drugs') AND ('coronavirus disease' OR 'sars-cov-2 infection' OR 'covid-19')
3. Search results：


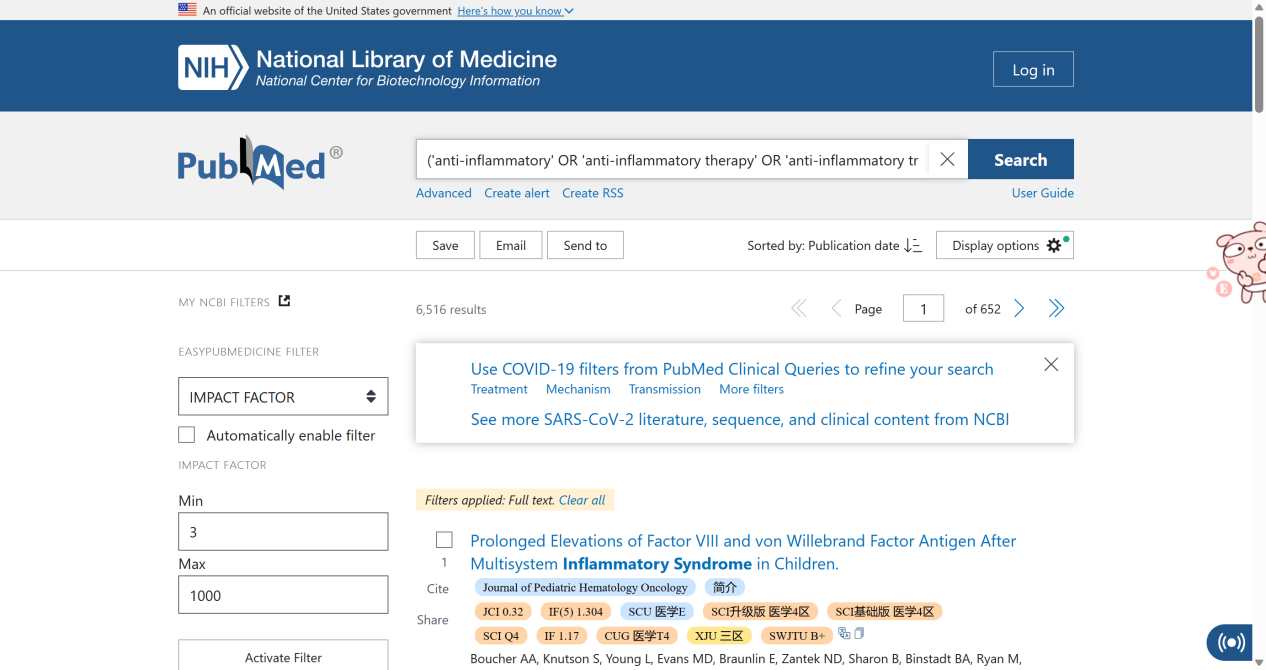


1. Web of Science：

（1）Search formula：(((TS=(Antiinflammatory)) OR TS=( Antiinflammatory therapy) OR TS=(Anti-inflammatory treatment) OR TS=(Anti-inflammatory drugs)) AND (TS=(Coronavirus disease) OR TS=(serious cold) OR TS=(SARS-Cov-2) OR TS=(Middle East Respiratory Syndrome )))

（2）Search results：


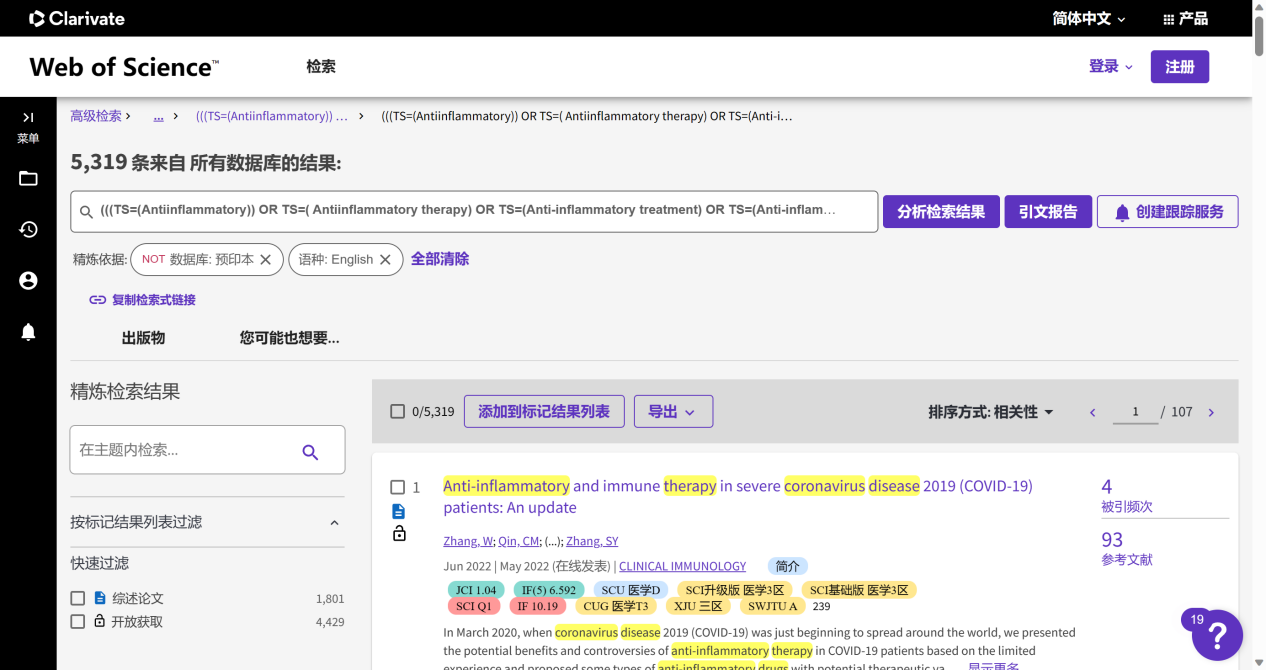


1. Embase and Medline：

（1）Search formula：('anti-inflammatory' OR 'anti-inflammatory therapy' OR 'anti-inflammatory treatment' OR 'anti-inflammatory drugs') AND ('coronavirus disease' OR 'sars-cov-2 infection' OR 'covid-19')

（2）Search results：


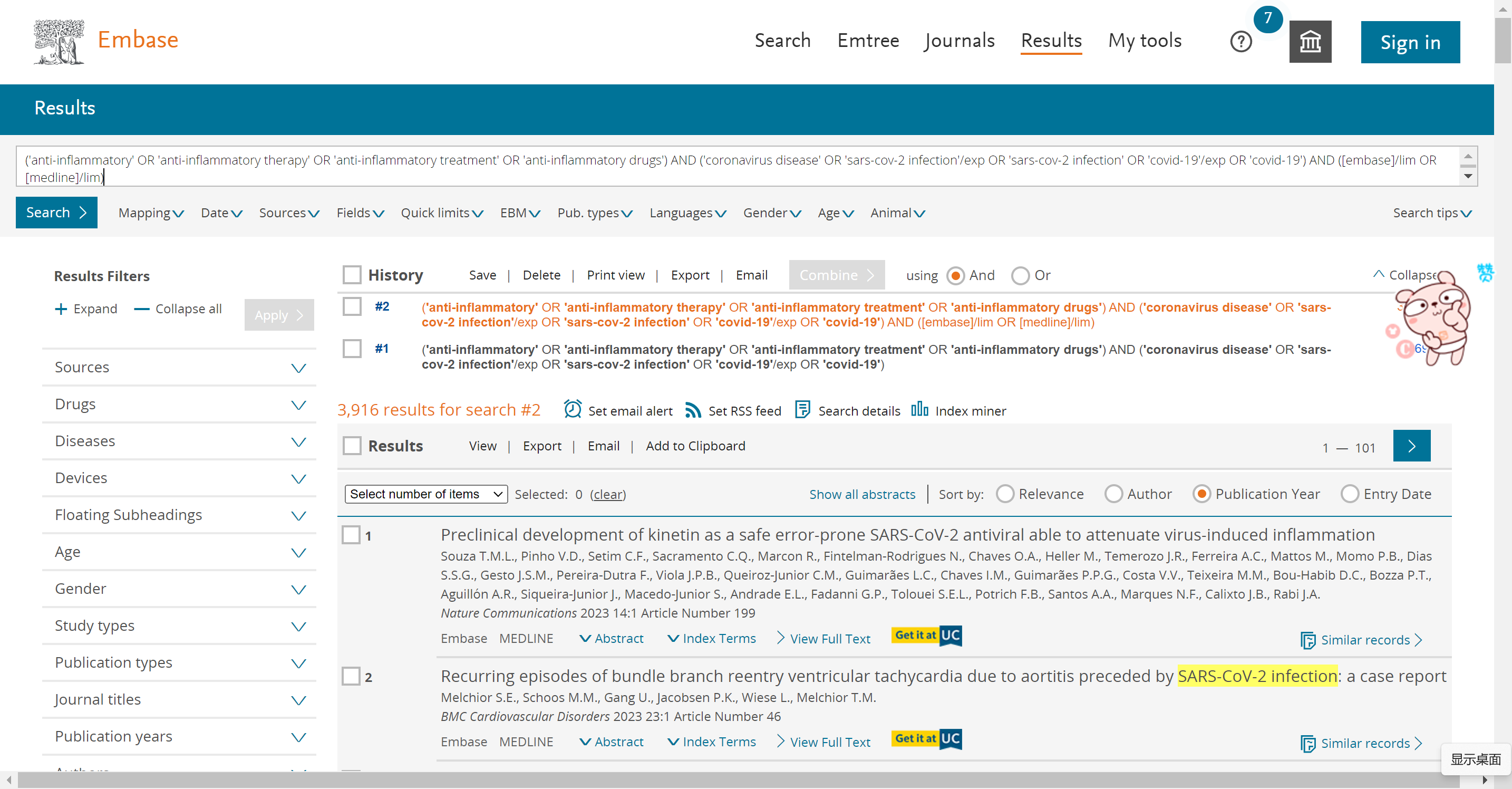


1. Cochrane Library：

（1）Search formula：('anti-inflammatory' OR 'anti-inflammatory therapy' OR 'anti-inflammatory treatment' OR 'anti-inflammatory drugs') AND ('coronavirus disease' OR 'sars-cov-2 infection' OR 'covid-19')

（2）Search results：


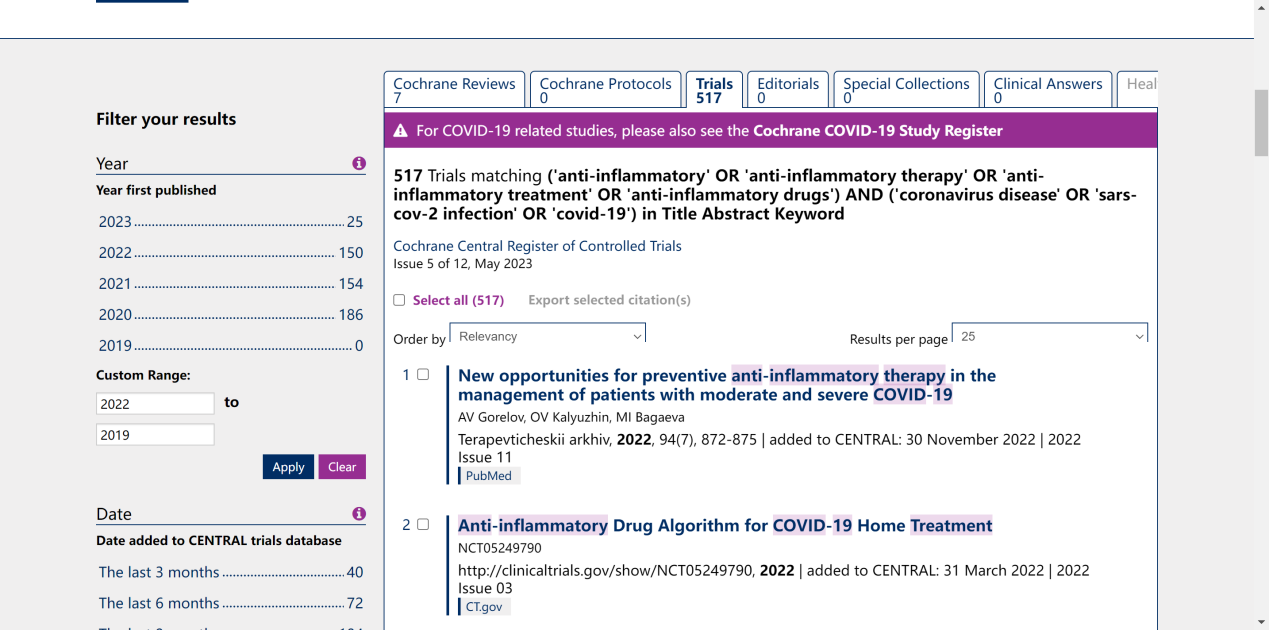

Supplement: Supplementary file 2 [file Data_Sheet_2.docx]
